# Mobile Technology Use Across Age Groups in Patients Eligible for Cardiac Rehabilitation: Survey Study

**DOI:** 10.2196/mhealth.8352

**Published:** 2017-10-24

**Authors:** Robyn Gallagher, Kellie Roach, Leonie Sadler, Helen Glinatsis, Julie Belshaw, Ann Kirkness, Ling Zhang, Patrick Gallagher, Glenn Paull, Yan Gao, Stephanie Ruth Partridge, Helen Parker, Lis Neubeck

**Affiliations:** ^1^ Charles Perkins Centre Sydney Nursing School University of Sydney Camperdown Australia; ^2^ Ryde Hospital Northern Sydney Local Health District Sydney Australia; ^3^ Manly Hospital Northern Sydney Local Health District Sydney Australia; ^4^ Royal North Shore Hospital Northern Sydney Local Health District Sydney Australia; ^5^ Hornsby Ku-ring-gai Hospital Northern Sydney Local Health District Sydney Australia; ^6^ St George Hospital South Eastern Sydney Local Health District Sydney Australia; ^7^ Charles Perkins Centre Sydney School of Public Health University of Sydney Sydney Australia; ^8^ Charles Perkins Centre Faculty of Health Sciences University of Sydney Sydney Australia; ^9^ School of Health and Social Care Edinburgh Napier University Edinburgh United Kingdom

**Keywords:** mobile phone, cell phone, digital divide, cardiac rehabilitation, cardiovascular disease

## Abstract

**Background:**

Emerging evidence indicates mobile technology–based strategies may improve access to secondary prevention and reduce risk factors in cardiac patients. However, little is known about cardiac patients’ use of mobile technology, particularly for health reasons and whether the usage varies across patient demographics.

**Objective:**

This study aimed to describe cardiac patients’ use of mobile technology and to determine variations between age groups after adjusting for education, employment, and confidence with using mobile technology.

**Methods:**

Cardiac patients eligible for attending cardiac rehabilitation were recruited from 9 hospital and community sites across metropolitan and rural settings in New South Wales, Australia. Participants completed a survey on the use of mobile technology devices, features used, confidence with using mobile technology, willingness and interest in learning, and health-related use.

**Results:**

The sample (N=282) had a mean age of 66.5 (standard deviation [SD] 10.6) years, 71.9% (203/282) were male, and 79.0% (223/282) lived in a metropolitan area. The most common diagnoses were percutaneous coronary intervention (33.3%, 94/282) and myocardial infarction (22.7%, 64/282). The majority (91.1%, 257/282) used at least one type of technology device, 70.9% (200/282) used mobile technology (mobile phone/tablet), and 31.9% (90/282) used all types. Technology was used by 54.6% (154/282) for health purposes, most often to access information on health conditions (41.4%, 117/282) and medications (34.8%, 98/282). Age had an important independent association with the use of mobile technology after adjusting for education, employment, and confidence. The youngest group (<56 years) was over 4 times more likely to use any mobile technology than the oldest (>69 years) age group (odds ratio [OR] 4.45, 95% CI 1.46-13.55), 5 times more likely to use mobile apps (OR 5.00, 95% CI 2.01-12.44), and 3 times more likely to use technology for health-related reasons (OR 3.31, 95% CI 1.34-8.18). Compared with the older group, the middle age group (56-69 years) was more than twice as likely to use any mobile technology (OR 2.42, 95% CI 1.27-4.59) and mobile technology for health-related purposes (OR 1.92, 95% CI 1.04-3.53). Participants who had completed high school were twice as likely to use mobile technology (OR 2.62, 95% CI 1.45-4.70), mobile apps (OR 2.05, 95% CI 1.09-3.84), and mobile technology for health-related reasons (OR 5.09, 95% CI 2.89-8.95) than those who had not completed high school. Associations were also present between participants living in metropolitan areas and mobile technology use (OR 1.07, 95% CI 1.07-4.24) and employment and mobile app use (OR 2.72, 95% CI 1.44-5.140).

**Conclusions:**

Mobile technology offers an important opportunity to improve access to secondary prevention for cardiac patients, particularly when modified to suit subgroups. High levels of mobile technology use and health motivation need to be harnessed for secondary prevention.

## Introduction

Cardiovascular disease (CVD) is a leading cause of death and disability globally [1]. Recurrence of cardiac events is common, causing frequent hospitalizations and high costs to the health system [2]. Secondary prevention is the key to limiting recurrence, yet patients struggle with initiating and maintaining the required behaviors [3]. An important evidence-based, cost-effective secondary prevention strategy is comprehensive cardiac rehabilitation (CR). Participation in CR reduces mortality and risk factors, as well as promotes recovery and quality of life [4,5]. Despite this, CR is underutilized, with less than one-third of eligible patients attending and dropout rates estimated at 25% [6]. A key factor contributing to poor CR participation is that delivery is in-person and offered at limited times and locations, so patients with limited resources, comorbidities, and other demands, such as caring roles, are unable to attend [7,8]. Technology, particularly, mobile devices that provide Internet access, offers a potential solution to reduce these barriers and improve access to secondary prevention strategies.

Advantages of mobile technologies for secondary prevention include timely patient education, real-time tracking of behavior, reminders, and prompts. Persuasive technology design and gaming principles can also be incorporated to promote key risk reduction across the life course [9,10]. Patients may also access health information and connect with health professionals and fellow cardiac patients more directly. Patients and health care providers may benefit from an increased capacity to compile, store, and deliver data, which may be used to assess and improve effectiveness. When mobile technologies are incorporated or offered as an alternative to traditional CR, improvements in multiple risk factors occur and mortality benefits have shown to be equal for both modes of delivery [11]. However, evidence regarding the benefits of specific mobile technology–based strategies for secondary prevention in cardiac patients is still evolving. Furthermore, implementation of these new strategies into practice is rare [12], in part because of lack of convincing evidence that cardiac patients are currently using mobile technology and the perceptions that the older age of this population will be a barrier.

### Mobile Technology Use and Age

Mobile technologies have advanced rapidly and their adoption has been widespread in developed countries, with seniors showing the fastest adoption rates [13]. However, age is frequently perceived as a critical and a potential barrier to technology engagement because age influences the opportunities people have had to develop familiarity, skills, and confidence with technology from their education and employment experiences [14]. Barriers and facilitators may also be idiosyncratic to a particular technology or functionality for an age group [15]. People who are currently aged between 50 and 70 years tend to have used computers, Internet, email, and various other technologies and features in their work and daily life but, perhaps, not a mobile phone. When this age group does use a mobile phone, they tend not to use all the features, such as apps, or may not do so confidently [15]. Whereas, people aged under 50 years tend to have been exposed to multiple technologies through education and employment; therefore, they are more likely to confidently use the full extent of mobile phone features, including schedulers, apps, and social media. In contrast, people aged 70 years and older generally use devices in a more passive way, such as using a mobile phone for voice calls and receiving texts [16,17]. This older subgroup will turn to computers and tablet devices for Internet use [15], in part, for the bigger screen because of visual impairment, and they tend to rely on younger people in areas where they are less confident, such as for setup and problem solving [17]. Therefore, the influence of the patient’s age is crucial to consider in any investigation of technology use for health [14].

### Mobile Technology Use in Cardiac Rehabilitation Patients

Research into cardiac patients’ engagement with mobile technology is in its infancy. Two studies were found that investigated technology use in CR patients of samples in New Zealand (n=74) [18] and Ireland and Belgium (n=298) [19]. The majority (97% [72/74] and 93.9% [280/298]) had a mobile phone, and mobile phone use was 38% (21/74) and 63.1% (188/298), respectively, with 74% (55/74) and 74.0% (220/298) of both samples accessing the Internet daily. Older patients were less likely to use mobile phone features or to be interested in Web-based CR programs [19]. The influence of education, employment, and confidence in mobile technology use was not assessed, and may have been significant, given that the samples from Ireland and Belgium were highly educated [19]. A more thorough understanding of the use of technology devices and functionalities across age groups of cardiac patients is needed. This knowledge will ensure that health technology interventions can be developed with an understanding of the subgroup for whom they are most likely to benefit and or modified to ensure that the attributes and requirements are suitable to the larger population of cardiac patients.

This study aimed to describe cardiac patients’ patterns of use of mobile technology and to determine the impact of age group after adjusting for education, employment, and confidence in mobile technology use.

## Methods

### Design and Patients

This multisite study involved a cross-sectional survey of cardiac patients, both in metropolitan settings (university [n=3] and community [n=3] hospitals) and rural settings (university [n=1] and community [n=2] hospitals), in New South Wales, Australia. Human research ethics approval was received from all institutions involved LNR/15 HAWKE/450.

Patients met inclusion criteria if they were: (1) current inpatients with a cardiac diagnosis and were eligible to be referred to CR, or (2) currently enrolled in a CR program, and (3) had sufficient understanding of the English language for consent and questionnaire processes. Patients with neurocognitive disorders and major visual impairment were excluded.

Sample size was calculated to be 250 patients, based on eight variables (gender, age group, home language English, education, marital/partner status, employment status, metropolitan or rural residence, confidence in mobile technology use) and on the basis of multiple regression analysis of technology engagement, and power was set at 80% and alpha=.05.

Current inpatients eligible for referral to and or patients currently attending CR were approached to participate in the study; once their consent was obtained, the survey was completed. Staff received training to ensure the survey process was standardized and remained present to assist if needed. A total of 296 patients were approached and 282 were recruited; reasons for refusal included not interested in being involved in research (n=6) and currently not using technology of any type and therefore not interested in this specific project (n=8).

### Data Collection

Technology engagement was assessed using a 20-item survey combining components of questionnaires developed by Edwards et al [20] for use and confidence-in-use and Illiger et al [21] for use of mobile technology. All of the following questions were in checklist format with tick-box responses for when the item applied. Questions related to whether participants currently used technology devices (computer, tablet, mobile phone, voice/text only phone, and activity trackers) and features that were regularly used (voice calls, text messages, email, Internet, Skype/Facetime, mobile apps, social media, scheduling, and information access). Participants were then asked separately to identify the devices they, (1) felt confident in using; (2) could easily learn; and (3) would like to learn to use. Additionally, participants were then asked to identify any health-related use of the Internet to (1) access information on health and heart conditions, treatments, medications, and lifestyle change and (2) communication with health professionals or other heart patients.

Confidence with technology use was modified from the original questionnaires to refer to technology overall [20]. However, pilot testing of this item indicated that participants focused primarily on using new programs. Therefore, an item was created that assessed confidence with technology use based on how quickly participants felt they could use a new program on any device (1=very quickly to 4=very slowly). A pilot test of the full survey was conducted on 15 cardiac patients to assess the appropriateness of format and understanding of survey items, minor modifications were then made to improve readability, accuracy, and specificity.

Sociodemographic data (age, gender, ethnicity, home language, education level achieved, marital/partnership status, and employment) and clinical details were collected to characterize the sample and include in the analyses [22], Patients who indicated they did not use any technology completed the sociodemographic and clinical details only.

### Statistical Analysis

Sociodemographic characteristics, engagement with different types of technology and functionalities were described using means, standard deviations (SD), frequencies, and percentages. Participants were grouped by age into categories of <56, 56-69, and >69 years to allow comparisons with the literature [14,16] and with reference to population level surveys of technology access and use [15,23]. The most relevant for the study context is the DeLoitte 2015 Australian technology survey, which categorized older Australians using a 68-year age threshold [15], and as the study recruited 1 year later than the report, 69 years of age was used. The final category was used to differentiate the age group for which technology was integral to their education and employment, in this case 56 years [24]. Comparisons between age groups were conducted using chi-square test for categorical variables and one-way analysis of variance followed by Tukey test for continuous variables. “Mobile technology” was defined as use of a mobile phone or tablet, and “health-related use” as Internet use to access health information or communicate with health professionals or other heart patients. The independent factors associated with mobile technology, mobile apps, and health-related Internet use were determined using simple linear regression analysis for each technology using the variables such as gender, age group, home language English, completed high school, marital/partner status, currently employed, metropolitan or rural resident, and overall confidence. All assumptions required for the linear regression analysis were met. The *P* value was set at .05 for all analyses, with Bonferroni correction to *P* value of .01 when multiple analyses occurred.

## Results

**Figure 1:**
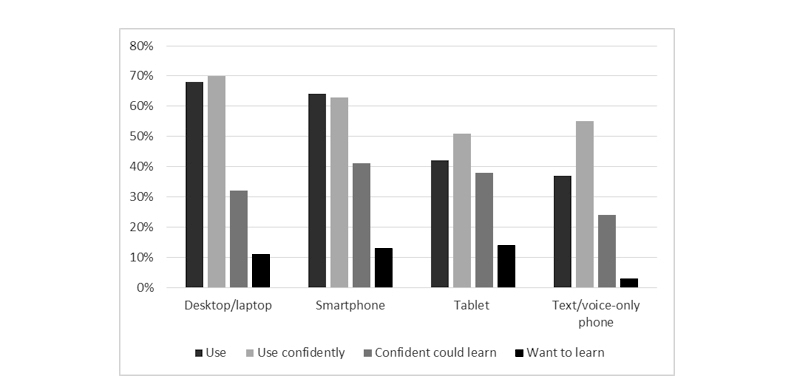
Use, confidence, and willingness to learn to use different technology devices.

The sample (n=282) had a mean age of 66.5 (SD 10.6, range 31-92) years, 72.0% (203/282) were male, and 79.1% (223/282) lived in a metropolitan area (Table 1). All patients had at least one cardiac diagnosis, the most common being percutaneous coronary intervention (33.3%, 94/282), myocardial infarction (22.7%, 64/282), and coronary artery bypass graft surgery (22.3%, 63/282). The majority (91.1%, 258/282) of participants currently used at least one type of technology, 70.9% (200/282) used mobile technology (mobile phone/tablet), and 31.9% (90/282) used all types. The most common single technology used was desktop/laptop computers (68.1%, 192/282) followed by mobile phones (63.8%, 180/282), mobile phones were also the device reported most often reported as being used confidently, correspondingly 69.9% (197/282) and 62.8% (177/282) (Figure 1). Mobile phones and tablets were the types of technology that if not currently used, participants most often felt confident they could learn to use (41.1%, 116/282 and 37.9%, 107/282, respectively) and wanted to learn to use (13.1%, 37/282 and 14.2%, 40/282, respectively). As age increased, participants were less likely to use any mobile technology (mobile phone/tablet) (overall and post hoc bivariate analyses *P*<.001), and overall confidence for technology use decreased significantly (overall and post hoc bivariate analyses *P*<.001) (Table 1).

**Table 1:** Sample characteristics and technology use of study participants compared for age category (N=282).

Characteristics		Age category (years)			*P* value, across ages
		<56, N=44	56-69, N=123	>69, N=115	
**Gender**					
	Male, n (%)	34 (77)	91 (74.0)	79 (68.7)	.52
Married/partnered, n (%)		33 (75)	82 (66.7)	69 (60.0)	.18
English primary language, n (%)		42 (95)	111 (90.2)	105 (91.3)	.79
Completed high school, n (%)		30 (68)	81 (65.9)	67 (58.3)	.36
Employed, n (%)		29 (65)	49 (39.8)	6 (5.2)	<.001
Metropolitan residence n (%)		35 (79)	96 (78.0)	94 (81.7)	.77
**Technology use**					
	Mobile technology^a^, n (%)	39 (88)	96 (78.0)	65 (56.5)	<.001^b^
	Mobile apps, n (%)	31 (70)	52 (42.3)	24 (20.9)	<.001^b^
	Health-related use, n (%)	32 (72)	74 (60.2)	49 (42.6)	.001^b^
Confidence (1-highest, 4-lowest), mean (SD)		2 (0.92)	2.41 (0.96)	2.67 (0.89)	.001^b^

^a^mobile phone or tablet.

^b^post hoc analyses all *P*<.01.

**Figure 2:**
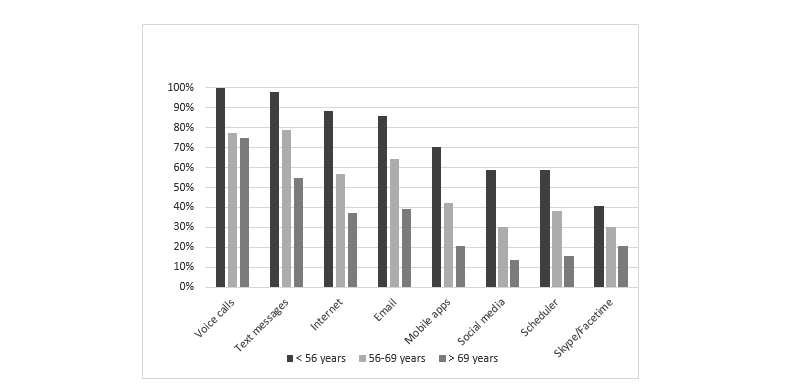
Use of technology features compared by age group.

Mobile technology (mobile phone/tablet) features used most often were voice calls (79.8%, 225/282), text messaging (70.6%, 199/282), sending email (55.3%, 156/282), using the Internet (52.1%, 147/282), and mobile apps (37.9%, 107/282). A small proportion (9.9%, 28/282) used all functionalities. With advancing age, every type of feature was used significantly less often, including mobile apps, with the exception of voice calls and Skype/Facetime (overall and post hoc bivariate analyses *P*<.001) (Figure 2,Table 1).

**Figure 3:**
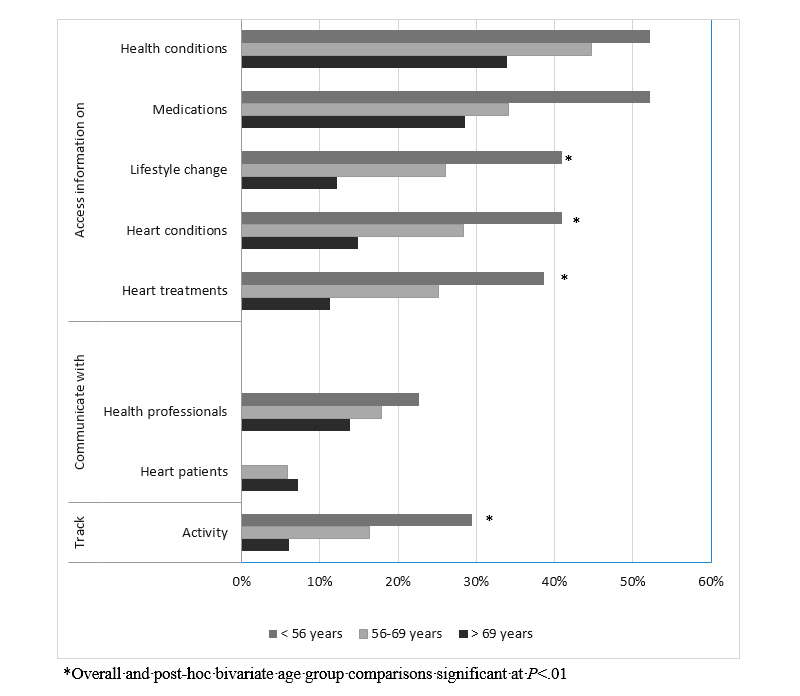
Health-related technology uses compared by age group.

Technology was used by 54.6% (154/282) for health purposes, which included accessing health information, and this occurred most often for health conditions (41.5%, 117/282) and medications (34.8%, 98/282). As the age of the sample increased, health-related use decreased significantly (overall and post hoc bivariate analyses *P*<.001) (Table 1), including accessing information related to lifestyle changes, heart conditions and treatments, and communicating with health professionals (overall and post hoc bivariate analyses *P*<.01) (Figure 3). In contrast, accessing information on health conditions and medication information did not alter significantly. Different patterns of use were observed (not statistically tested) across age groups as the gap between mobile technology device use and the use of the device features, such as apps, was much larger in the two older groups (>69 years: mobile technology use of 56.5% [65/115] vs app use of 20.9% [24/115]; 56-69 years: mobile technology use of 78.0% [96/123] vs app use of 42.3% [52/123]), than in the youngest group (<56 years: mobile technology use of 88% [39/44] vs app use of 70% [31/44]) (Table 1). This gap was also present in health-related technology use but was much smaller and similar across age groups (>69 years: mobile technology use of 56.5% [65/115] vs health-related use of 42.6% [49/115]; 56-69 years: mobile technology use of 78.0% [96/123] vs health-related use of 60.2% [74/123]; and <56 years: mobile technology use of 88% [39/44] vs health-related use of 72% [32/44]).

Age had an important independent association with mobile technology use after adjusting for education, and employment and other important variables (Table 2). Compared with the oldest age group, the youngest age group was at least four times more likely to use any mobile technology (odds ratio [OR] 4.45, 95% CI 1.46-13.55), 5 times more likely to use any mobile apps (OR 5.0, 95% CI 2.01-12.44), and 3 times more likely to use mobile technology for health-related reasons (OR 3.31, 95% CI 1.34-8.18). This association was evident but less pronounced when the middle age group was compared with the oldest age group, with participants more than twice as likely to use any mobile technology (OR 2.42, 95% CI 1.27-4.59) and mobile technology for health-related purposes (OR 1.92, 95% CI 1.04-3.53). Education was also important, with participants who had completed high school being much more likely to use any mobile technology (OR 2.62, 95% CI 1.45-4.70), mobile apps (OR 2.05, 95% CI 1.09-3.84), or to use mobile technology for health-related reasons (OR 5.09, 95% CI 2.89-8.95), rather than those who had not completed high school. Living in metropolitan areas increased the likelihood of any mobile technology use (OR 2.13, 95% CI 1.07-4.24), and employment increased the likelihood of using any apps (OR 2.72, 95% CI 1.44-5.14).

**Table 2:** Factors independently associated with mobile (mobile phone/tablet) technology use.

Characteristics		Mobile phone/tablet		Mobile apps		Health-related	
		OR^a^ (95% CI)	*P* value	OR (95% CI)	*P* value	OR (95% CI)	*P* value
**Age**							
	<56 years vs >69 years	4.45 (1.46-13.55)	.009	5.00 (2.01-12.44)	.001	3.31 (1.34-8.18)	.01
	56-69 years vs >69 years	2.42 (1.27-4.59)	.007	1.79 (0.93-3.45)	.08	1.92 (1.04-3.53)	.04
Completed high school		2.62 (1.45-4.70)	.007	2.05 (1.09-3.84)	.02	5.09 (2.89-8.95)	<.001
Metropolitan residence		2.13 (1.07-4.24)	.03	1.41 (0.65-3.04)	.39	1.63 (0.82-3.23)	.16
Employed		1.94 (0.87-4.33)	.12	2.72 (1.44-5.14)	.002	1.35 (0.69-2.59)	.39
**Gender**							
	Male	1.66 (0.87-3.18)	.12	0.61 (0.32-1.14)	.12	0.67 (0.37-1.22)	.19

^a^OR: odds ratio.

## Discussion

### Principal Findings

This study provides evidence that majority of patients eligible for or already attending CR, use mobile technologies such as mobile phones or tablets, providing the first evidence for the feasibility of using these technologies as an important alternative for delivering secondary prevention in more diverse samples. The study results contribute to the currently limited evidence that technology use is also very common when the sample is more diverse, including lower education and language backgrounds. Use of texting, Internet, and email were particularly high across all age groups. However, age and education were important influences in technology use and confidence of use. Younger and more educated patients were more likely to use mobile technology and to do so for health reasons, as well as to use apps, especially, if they were employed. Younger patients were also more confident in technology use.

Mobile technology use was high and comparable to the other limited studies in cardiac patients, despite the sample having much lower education levels. Mobile phone use (63.8%, 180/282), was similar to reports from Ireland and Belgium 63.1% (188/298), [19] but much higher than a sample from New Zealand 38% (21/74) [18]. An earlier recruitment year for the New Zealand sample may have contributed to this variation, despite being only 3 to 4 years, given the rapid penetration of mobile phones into the market and uptake of mobile technology in older groups such as cardiac patients [15]. Furthermore, this study identifies that the majority (54.6%, 154/282) of patients eligible for CR are using mobile technologies for health-related purposes [19], which was higher than reports from studies of general patient samples [16,21]. Common health-related uses included accessing health information, communicating with health professionals, and the use of activity-tracking devices [19]. It is important to capitalize on these health-related motivations given that a recent systematic review suggests that mobile health (mHealth) interventions can improve cardiovascular-related lifestyle behaviors and disease management in a way that is scalable to the public health level [25]. It is also important to acknowledge that cardiac patients use multiple sources of informational and behavioral support for their health and to include these aspects in patient education and recommend credible and trustworthy sources [9] would be helpful. Identification of any subgroups of users within cardiac patients and insight into associated differences within these subgroups of users is essential to the process.

Age was an important defining factor in cardiac patients’ engagement with mobile technology, including for health-related purposes. This study adds to existing findings that a “digital divide” is present in mobile technology access and use for health reasons, and it also occurs in patients with cardiac conditions [14,16,26]. These previous studies proposed that the divide is the result of the relative presence of opportunities provided by education and employment that vary with age. This study is the first to identify that education and employment are indeed important, but the effect of age is also important and is independent of these aspects. Rather than a digital divide in mobile technology use created by age, education and employment, for cardiac patients the three age-defined groups identified by DeLoitte [15] proved accurate and reflected similarities to those identified by LeRouge et al [14]. For instance, younger individuals (<56 years) were highly engaged with mobile technology, using multiple devices and interactive features, such as apps, and frequently doing so for health. More than half of this group accessed online health and medication information, and more than a third accessed lifestyle and cardiac-related information and used trackers for their activity. The middle-aged group defined by LeRouge et al [14] as the Baby Boomer group (56-69 years) was also highly engaged with mobile technology, but their use was more narrowly focused, being far less likely to include interactive functionalities such as apps or trackers or to use mobile technology for health reasons. On the other hand, the oldest group (>69 years) were much less likely to be using mobile technology in all respects [14]. As a consequence, when mHealth interventions are developed, efforts should be made to ensure older patients are not accidentally excluded.

Aside from a lower likelihood of experience with mobile devices, aging is also accompanied by important changes in visual acuity and manual dexterity, which limits the potential use of small-screen devices [17]. Furthermore, the influence of older people’s social group, including their peers, can limit motivation [27]. However, people of all ages can be taught to use technology, and so, although usage is less, benefits may still be obtained, particularly when larger screen devices, such as tablets, are used [17]. Therefore, age continues to be an important consideration in the development and targeting of mobile technology–based interventions for secondary prevention. However, this effect is likely to be rapidly diluted in the coming years, given the rapidly changing technology and communication landscape [15].

Education is another important factor to consider, potentially because it is an indicator of socioeconomic status and inequality generally. This study found that participants who had completed high school were at least five times more likely to be using mobile technology for health reasons than those not completing high school, which is consistent with a national survey on eHealth use in the United States [28]. In that survey of 2358 adult cancer patients, respondents who had not completed high school were less likely than even the lowest income participants to use the Internet/mobile phone for health reasons [28]. However, other idiosyncrasies in technology use were evident in that study, with the least educated and oldest males the least likely to engage in any mobile technology for health and younger females more likely to use social media for health. In this study, gender was not associated with mobile technology use in any respect. However, the Kontos et al [28] study highlights the need for a detailed understanding of the feasibility and acceptability of mobile technologies and features before developing online health-related interventions [29]. There is also an imperative to ensure when mobile technology is used for health that the information and support accessed is accurate and appropriate to their condition and circumstances. When these measures have been used for online secondary prevention programs, uptake has been high and the intervention effective [30,31]. However, these examples are limited and further work is required, particularly to keep pace with rapidly changing technologies and features. Indeed, given the rapid adoption of technology by older people and the inevitable advancing age of the digital generation, there is an imperative for regular reassessment of health technology usage patterns [23].

### Limitations

While the study recruited participants from multiple diverse locations, the sample may not represent all cardiac patients eligible for CR. The survey used to collect data was developed and modified based on previous studies and while pilot-tested in the relevant population, it has not been tested previously. The age categories chosen were based on relevant cut-points from the literature on technology use, resulting in unequal sample sizes, particularly for the youngest age group. If equal sized age groups were used, this would not reflect digital habits identified in the literature. The question used to assess overall confidence with technology requires further development to ensure a more comprehensive understanding of confidence with technology, particularly in relation to individual technology features.

### Conclusions

This study identifies that mobile technology use in cardiac patients is at a high level, providing an important strategy for delivering secondary prevention, which should be harnessed. Furthermore, mobile technology offers an important opportunity to improve access to secondary prevention and enhance CR programs, particularly for younger patients for whom time and work pressures prove a barrier to participation. However, when developing mobile technology–based interventions, care must be taken not to presume that interventions demonstrated as applicable to younger age cardiac patients will also be directly applicable to older age patients.

## Abbreviations

CR: cardiac rehabilitation
CVD: cardiovascular disease
eHealth: electronic health
mHealth: mobile health
OR: odds ratio
SD: standard deviation
